# FTY720 Reduces the Biomass of Biofilms in *Pseudomonas aeruginosa* in a Dose-Dependent Manner

**DOI:** 10.3390/antibiotics13070621

**Published:** 2024-07-04

**Authors:** Abdurahman A. Niazy, Rhodanne Nicole A. Lambarte, Terrence S. Sumague, Mary Grace B. Vigilla, Najla M. Bin Shwish, Ranan Kamalan, Eid Khulaif Daeab, Nami M. Aljehani

**Affiliations:** 1 Department of Oral Medicine and Diagnostic Sciences, College of Dentistry, King Saud University, Riyadh 11545, Saudi Arabia; 2 Molecular and Cell Biology Laboratory, Prince Naif bin AbdulAziz Health Research Center, College of Dentistry, King Saud University Medical City, King Saud University, Riyadh 11545, Saudi Arabia; rlambarte@ksu.edu.sa (R.N.A.L.); tsumague@ksu.edu.sa (T.S.S.); nbinshiwsh@ksu.edu.sa (N.M.B.S.); 3 Research Center, College of Dentistry, King Saud University, Riyadh 11451, Saudi Arabia; 4 Department of Clinical Laboratory Science, College of Applied Medical Sciences, King Saud University, Riyadh 11433, Saudi Arabia

**Keywords:** biofilm biomass, biomass reduction, *Pseudomonas aeruginosa*, fingolimod, FTY720

## Abstract

*Pseudomonas aeruginosa*, a nosocomial pathogen, has strong biofilm capabilities, representing the main source of infection in the human body. Repurposing existing drugs has been explored as an alternative strategy to combat emerging antibiotic-resistant pathogens. Fingolimod hydrochloride (FTY720), an immunomodulatory drug for multiple sclerosis, has shown promising antimicrobial effects against some ESKAPE pathogens. Therefore, the effects of FTY720 on the biofilm capabilities of *Pseudomonas aeruginosa* were investigated in this study. It was determined that FTY720 inhibited the growth of *P. aeruginosa* PAO1 at 100 µM. The significant reduction in PAO1 cell viability was observed to be dose-dependent. Additional cytotoxicity analysis on human cell lines showed that FTY720 significantly reduced viabilities at sub-inhibitory concentrations of 25–50 µM. Microtiter assays and confocal analysis confirmed reductions in biofilm mass and thickness and the cell survivability ratio in the presence of FTY720. Similarly, virulence production and biofilm-related gene expression (*rhlA*, *rhlB*, *pilA*, *pilI*, *fliC*, *fliD* and *algR*) were determined. The results demonstrate that pigment production was affected and quantitative real-time PCR analysis showed a variable degree of reduced gene expression in response to FTY720 at 12.5–50 µM. These findings suggest that FTY720 could be repurposed as an alternative antibiofilm agent against *Pseudomonas aeruginosa*.

## 1. Introduction

Biofilms are an organized, complex group of microorganisms living safely within a self-produced matrix of extracellular polymeric substances that adhere to various surfaces [1,2]. Biofilm-related infections are associated with approximately 65–80% of global chronic infections involving a wide range of diseases [2,3,4]. As the pathogenic microbes inside biofilms are highly resistant to antimicrobials, this makes related infections difficult to control and treat [2]. The World Health Organization (WHO) identified the six ESKAPE pathogens (*Enterococcus faecium*, *Staphylococcus aureus*, *Klebsiella pneumoniae*, *Acinetobacter baumannii*, *Pseudomonas aeruginosa*, and *Enterobacter* spp.) that display extensive multidrug-resistance potential and are responsible for most nosocomial infections worldwide [5].

Due to the rapid emergence of antibiotic resistance and the slow pace of development for newer antibiotics, repurposing commercially available drugs for alternative clinical applications such as antimicrobials is being explored [6,7]. Drug repurposing is a strategy of identifying new therapeutic uses for existing drugs and medications, and further offers several advantages over the conventional drug discovery process, such as well-established toxicity profiles and reduced costs and risks [8,9]. The ability of pre-approved drugs to inhibit bacterial biofilm formation capabilities has led to the exploration of other unconventional compounds for potential use in various medical applications to overcome the increasing threat of antibiotic resistance [10,11]. Another advantage of this approach is that the medications that are screened for repurposing are those which are routinely prescribed for chronic diseases such as multiple sclerosis and cancer [12,13].

**Figure 1:**
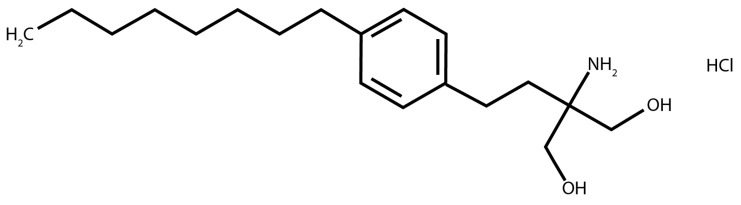
Chemical structure of fingolimod hydrochloride.

Fingolimod hydrochloride (FTY720; Figure 1) is a synthetic compound derived from myriocin, which is a secondary metabolite extracted from the fungus *Isaria sinclairii*, and is the first oral immunomodulatory drug for relapsing multiple sclerosis [14,15,16]. FTY720 prolongs allograft transplant survival in numerous models by inhibiting lymphocyte emigration from lymphoid organs by down-regulating the sphingosine-1-phosphate receptor (S1PR) [17,18]. Studies suggest that the therapeutic benefit of FTY720 treatment reduces the severity of neuroinflammatory-mediated demyelination by preventing neuronal damage and limiting the access of autoaggresive lymphocytes to the central nervous system [19]. 

The antimicrobial effects of FTY720 on other bacteria such as *A. baumannii*, *S. aureus*, and *E. coli* have recently been studied, increasing the potential for repurposing this drug to reduce bacterial pathogenicity and biofilm formation [20,21,22]. It was also shown that FTY720 had antifungal activity against the yeast *Candida albicans* in vitro and in a mouse candidiasis model [23,24]. Similarly, when analyzing its effects on methicillin-resistant *Staphylococcus aureus* (MRSA), FTY720 treatment attenuated the effects of MRSA-induced acute lung injury [25]. It was also proven that FTY720 prevented the dissemination of chlamydia in a mouse model [26]. Other than its direct effects on inhibiting microbial growth, it was also shown that FTY720 impaired mucosal immunity response and induced clearance of *Citrobacter rodentium* in an immunocompetent mouse model [27]. Another study determined that fingolimod had substantial antibiofilm properties and synergistic antibacterial potential for colistin in treating *K. pneumoniae* infections [28]. 

Despite the antimicrobial potential of FTY720, very little work has been carried out on its effects on biofilms and the pathogenicity of *Pseudomonas aeruginosa*. This opportunistic bacterium is notorious for forming strong biofilms which play a major role in its virulence [29]. Virulence factors produced by *P. aeruginosa* play important roles during host–cell invasion, such as the production of pyocyanin and pyoverdine pigments [30,31]. Pyocyanin is a blue-green pigmented, redox-active phenazine compound that generates reactive oxygen intermediates [30]. Additionally, *P. aeruginosa* produces pyoverdine, a fluorescent green-yellow siderophore. By facilitating iron acquisition, promoting bacterial growth, and stimulating the host immunological response, pigments play a significant role in the pathogenesis of *P. aeruginosa* infections [31]. In addition, the microorganism has specialized motility, which is necessary for its ability to attach and aggregate to surfaces or patient devices [32]. Many genes have been determined to be involved in the cross-regulation between biofilm formation and motility. *pilA* and *pilI* are genes involved in pilus formation, which is implicated in both motility and biofilm formation [33]. *fliC* and *fliD* are genes involved in flagella formation, which is important for bacterial swimming and biofilm production. *algR* is a gene involved in alginate formation, which is an important glycoprotein produced in *Pseudomonas* species [34]. Rhamnolipids are glycolipid biosurfactants produced by *P. aeruginosa* and are controlled by the *rhl* gene family, mainly *rhlA* and *rhlB* [35]. *P. aeruginosa* PAO1 is one of the most frequently used reference strains and is a model for biofilm studies since it has a complete genome sequence available in databases [36,37].

Based from these, this study aimed to assess the antibiofilm and antibacterial potential of fingolimod hydrochloride against *P. aeruginosa* and investigate its preliminary mechanisms of action. 

## 2. Results

### 2.1. Resazurin-Based Turbidometric Assay for Minimum Inhibitory Concentration (MIC) Determination

**Figure 2:**
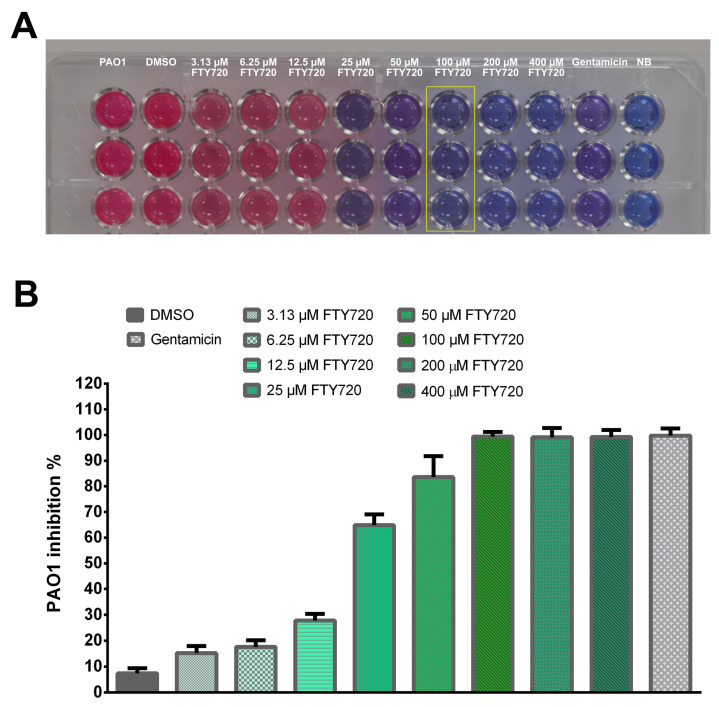
(**A**) Resazurin-based MIC determination of FTY720 against *P. aeruginosa* PAO1. (**B**) Inhibition percentages on the bacterial growth of *P. aeruginosa* upon FTY720 exposure. Data are expressed as mean ± SD (n = 9).

FTY720 treatment at 100 µM showed minimum inhibitory effects against PAO1 (Figure 2A). The DMSO concentration used in the experiment had no inhibitory effects on Pseudomonas aeruginosa. The percentages for PAO1 growth inhibition were 15.23 ± 2.71%, 17.63 ± 2.53%, 27.76 ± 2.58%, 64.86 ± 4.22%, 83.60 ± 8.20%, 99.38 ± 1.83%, 99.12 ± 3.58%, and 99.23 ± 2.71%, respectively, for concentrations of 3.13, 6.25, 12.5, 25, 50, 100, 200, and 400 µM FTY720 (Figure 2B).

### 2.2. Cytotoxicity

**Figure 3:**
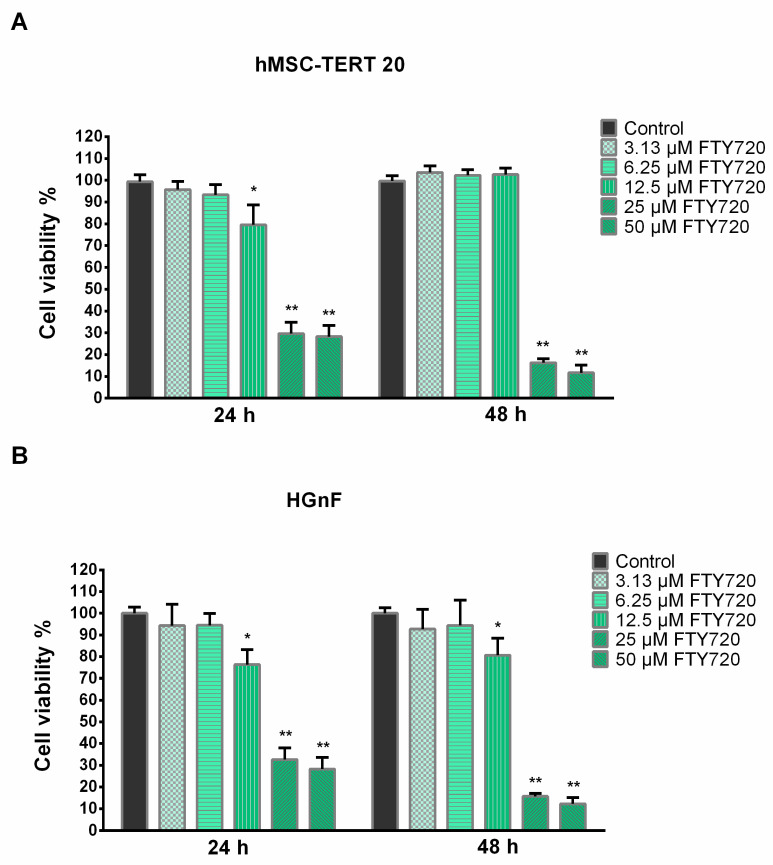
Effects on the viability of (**A**) immortalized human bone marrow mesenchymal stem cells and (**B**) human gingival fibroblasts exposed to FTY720 treatment. Data are presented as mean ± SD (n = 9); * *p* < 0.05; ** *p* < 0.01.

To further investigate the toxicity profile of FTY720 on human cell lines, the cytotoxicity of FTY720 concentrations related to MIC determination was evaluated on telomerized-human mesenchymal stem cells (hMSC-TERT 20) and human gingival fibroblast (HGnF) cell lines. FTY720 showed no significant cytotoxic effects for both cell lines at 3.13 and 6.25 µM at both exposure periods. At 12.5 µM, the hMSC-TERT 20 cells showed a significant reduction in cell viability after 24 h, while the viabilities of HGnF cells were significantly reduced at both 24 and 48 h. Meanwhile, FTY720 showed significant cytotoxic effects on both cell lines at 25 and 50 µM after 24 and 48 h of exposure. The cell viabilities for hMSC-TERT 20 cells were 95.76 ± 3.78%, 93.38 ± 4.66%, 79.58 ± 9.22%, 29.68 ± 5.24% and 28.26 ± 5.17%, respectively, for concentrations of 3.13, 6.25, 12.5, 25, and 50 µM FTY720 after 24 h, and 103.62 ± 2.94, 102.28 ± 2.64%, 102.65 ± 2.98%, 16.29 ± 1.80%, and 11.69 ± 3.51% after 48 h (Figure 3A). HGnF showed viability percentages of 94.35 ± 9.79%, 94.53 ± 5.32%, 76.32 ± 6.91%, 32.62 ± 5.40%, and 28.23 ± 5.40%, respectively, for 3.13, 6.25, 12.5, 25, and 50 µM of FTY720 after 24 h of exposure and 92.73 ± 9.04%, 94.39 ± 11.72%, 80.65 ± 7.91%, 15.71 ± 1.39%, and 12.27 ± 2.93% after 48 h (Figure 3B). 

### 2.3. Growth Curve and Metabolic Activities

**Figure 4:**
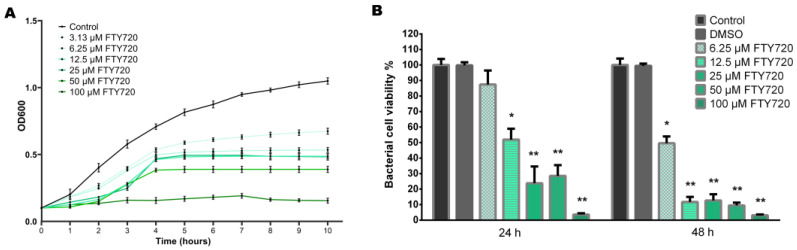
(**A**) Effects of FTY720 treatment on the growth of *P. aeruginosa* PAO1. (**B**) Effects on the bacterial cell viability of *P. aeruginosa* upon FTY720 exposure. Data are expressed as mean ± SD (n = 6); * *p* < 0.05; ** *p* < 0.01.

The growth curve in Figure 4A shows slower growth for bacteria exposed to FTY720 than the untreated bacterial cultures. The results demonstrate that FTY720 with an MIC of 100 µM inhibited the growth of *P. aeruginosa* PAO1. They also show that cells exposed to FTY720 at 3.13–50 µM had a longer log phase and displayed a slower rate of reaching the stationary phase than the control PAO1 cells. However, treatment at these tested concentrations did not have direct antimicrobial properties for planktonic *P. aeruginosa* cells.

The resazurin-based assay indicated a dose-dependent decrease in *P. aeruginosa* viability, with the highest reduction in cell activity observed at a fingolimod concentration of 100 µM (Figure 4B). Nevertheless, a 50% reduction in the bacterial viability of *P. aeruginosa* was observed for 12.5 µM FTY720 after 24 h when compared to the control cells. At 24 h, the bacterial viability percentages were 87.45 ± 12.06%, 50.02 ± 7.16%, 23.79 ± 6.53%, 28.54 ± 6.85%, and 5.83 ± 1.28% for the 6.25, 12.5, 25, 50, and 100 µM FTY720 treatments, respectively. Continuous culturing in the presence of FTY720 for 48 h showed significantly increased inhibition in bacterial cell viability for all concentrations. Similarly, at 48 h, PAO1 cell viabilities decreased to 49.56 ± 10.78%, 11.72 ± 8.00%, 12.68 ± 9.82%, 9.50 ± 4.35%, and 4.88 ± 1.23% for 6.25, 12.5, 25, 50, and 100 µM FTY720, respectively. 

### 2.4. Biofilm Formation

**Figure 5:**
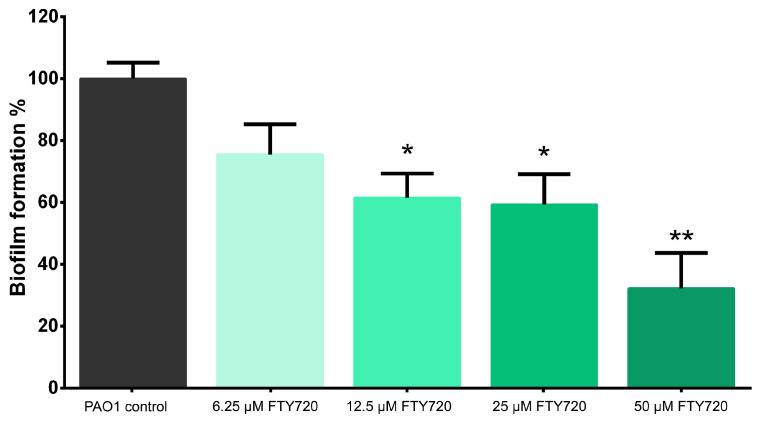
Effects of the different concentrations of FTY720 on the biofilm formation of *P. aeruginosa* PAO1 at 24 h. Data are expressed as mean ± SD (n = 6); * *p* < 0.05; ** *p* < 0.01.

The increase in FTY720 drug concentration decreased the biofilm formation capabilities in a dose-dependent manner, wherein the 50 µM treatment showed the maximal inhibition of biofilm formation (Figure 5). The percentage of biofilm formation in comparison to the growth control sample was as follows: 75.56 ± 9.78%, 61.53 ± 7.85%, 59.39 ± 9.78%, and 32.27 ± 11.40% for 6.25, 12.5, 25, and 50 µM FTY720, respectively.

### 2.5. Biomass in Biofilms

**Figure 6:**
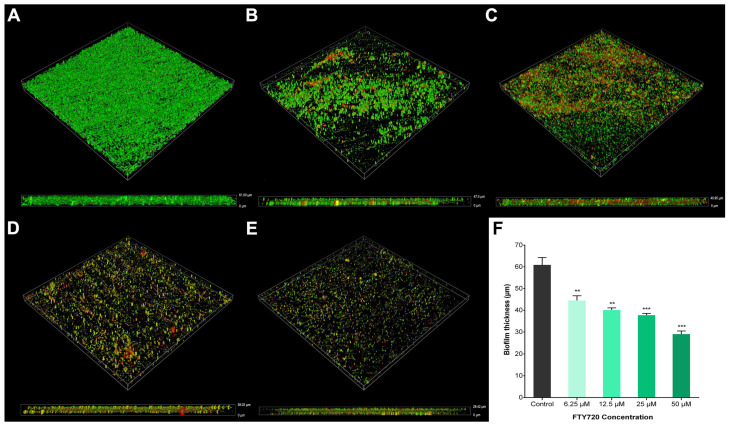
Disruptive effects of FTY720 on *P. aeruginosa* biofilms. Representative images of PAO1 biofilms after treatment for 48 h with FTY720 using CLSM. Biofilms of (**A**) untreated control and treatment with (**B**) 6.25, (**C**) 12.5, (**D**) 25, and (**E**) 50 µM FTY720. (**F**) Biofilm thickness measurements. Data are expressed as mean ± SD (n = 6); ** *p* < 0.01; *** *p* < 0.001.

The thickness of the disrupted biofilms was measured using CLSM and quantified using image slice z-stack analysis. It was found that PAO1 biofilm cells adhered on control coverslips were covered with an abundance of polysaccharides with a biomass thickness of 62.88 ± 1.38 µm, whereas 50 µM FTY720 caused the largest reduction in biofilm thickness, reducing it to 29.02 ± 2.53 µm. Exposure to 6.25 µM FTY720 led to a reduction in thickness to 44.52 ± 4.32 µm, while 12.5 µM FTY720 produced a biofilm thickness of 40.07 ± 1.79 µm and 25 µM resulted in a biofilm thickness of 37.75 ± 1.41 µm. All concentrations used for treatment with FTY720 showed a statistically significant reduction in biofilm thickness compared to the untreated control (Figure 6).

### 2.6. Antibacterial Activity

**Figure 7:**
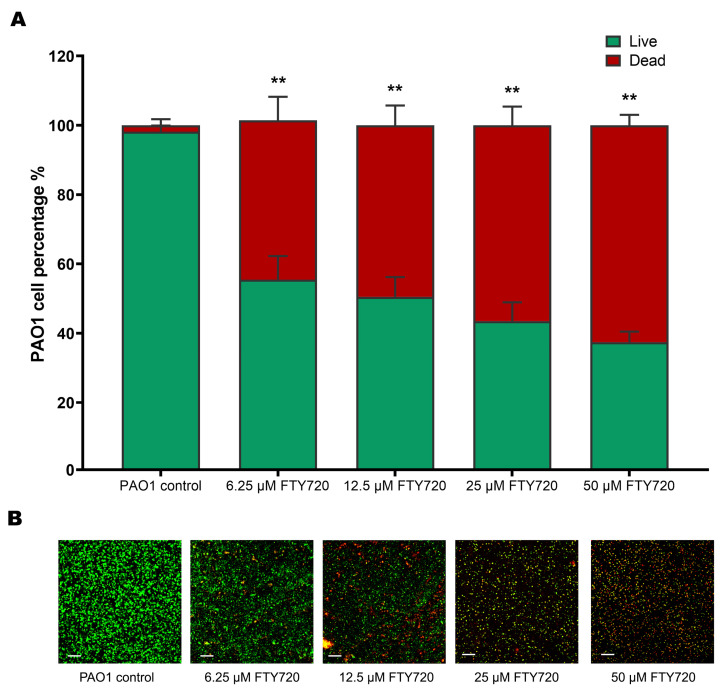
Effects on bacterial biofilm inhibition using the LIVE/DEAD assay with confocal laser scanning microscopy after 48 h of treatment with FTY720. Live (green) and dead (red) biofilm cells of PAO1. (**A**) PAO1 percentages and (**B**) representative images for control, 6.25, 12.5, 25, and 50 µM FTY720 exposure, respectively. Data are expressed as mean ± SD (n = 6); ** *p* < 0.01. Scale bar: 50 µm.

The 50 µM FTY720 concentration induced the highest percentage of cell death in comparison to the other concentrations, with 59.32 ± 2.74%, whereas 6.25, 12.5, and 25 µM resulted in cell death percentages of 44.54 ± 6.82%, 49.50 ± 5.70%, and 54.06 ± 4.61%, respectively (Figure 7). 

### 2.7. Pigment Production

**Figure 8:**
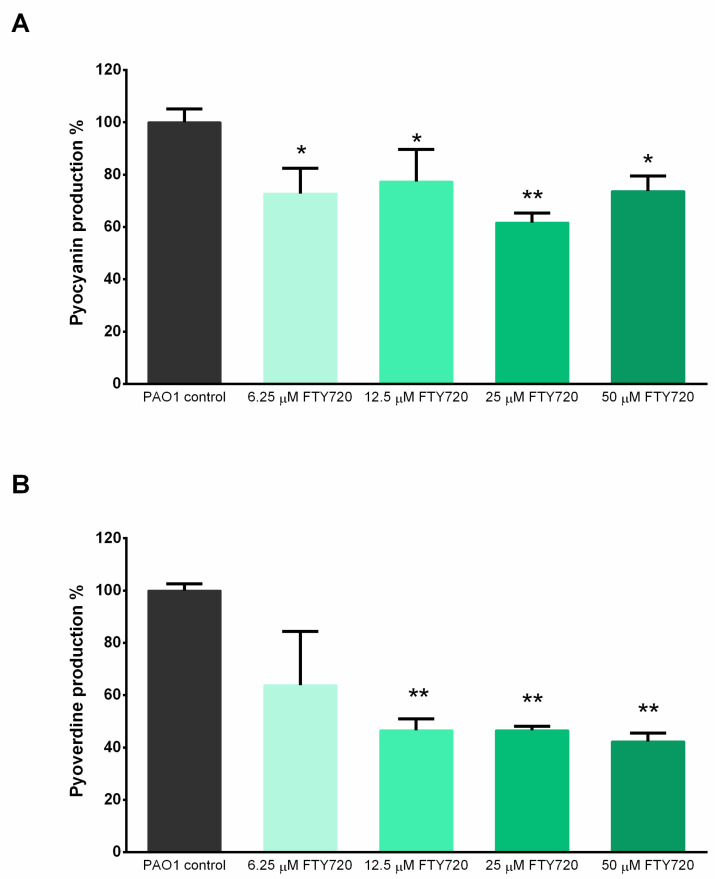
(**A**) Effects on the pyocyanin production of *P. aeruginosa* after treatment with FTY720 compared to control cultures. (**B**) Effects on the biosynthesis of pyoverdine siderophores by *P. aeruginosa* after exposure to FTY720. Data are expressed as mean ± SD (n = 6); * *p* < 0.05; ** *p* < 0.01.

Our results demonstrate that 25 µM FTY720 exposure significantly inhibited and reduced the *P. aeruginosa* virulence of pyocyanin production. Pyocyanin pigmentation was affected in a statistically significant manner after exposure to various FTY720 concentrations (Figure 8A). The concentration of 6.25 µM decreased pyocyanin production to 72.78 ± 9.75% of the level of the control, while 12.5 and 25 µM resulted in the production of pyocyanin reducing to 77.39 ± 12.26% and 61.69 ± 3.64% of the level of the control. Meanwhile, 50 µM treatment reduced pigment production to 73.66 ± 5.86% of the control level.

On the other hand, *P. aeruginosa* showed a significant decrease in pyoverdine production as a result of being exposed to various FTY720 concentrations. Treatment with 6.25 µM FTY720 reduced pyoverdine production to 63.89 ± 3.54% of the level of the control, 12.5 µM reduced it to 46.88 ± 2.87% of the level of the control, whereas 25 µM resulted in it reducing to 46.51 ± 7.70% of the control level. Overall, 50 µM FTY720 induced the most significant reduction in pyoverdine production, to 42.31 ± 5.66% of the control level (Figure 8B). 

### 2.8. Expression of Biofilm-Relevant Genes

**Figure 9:**
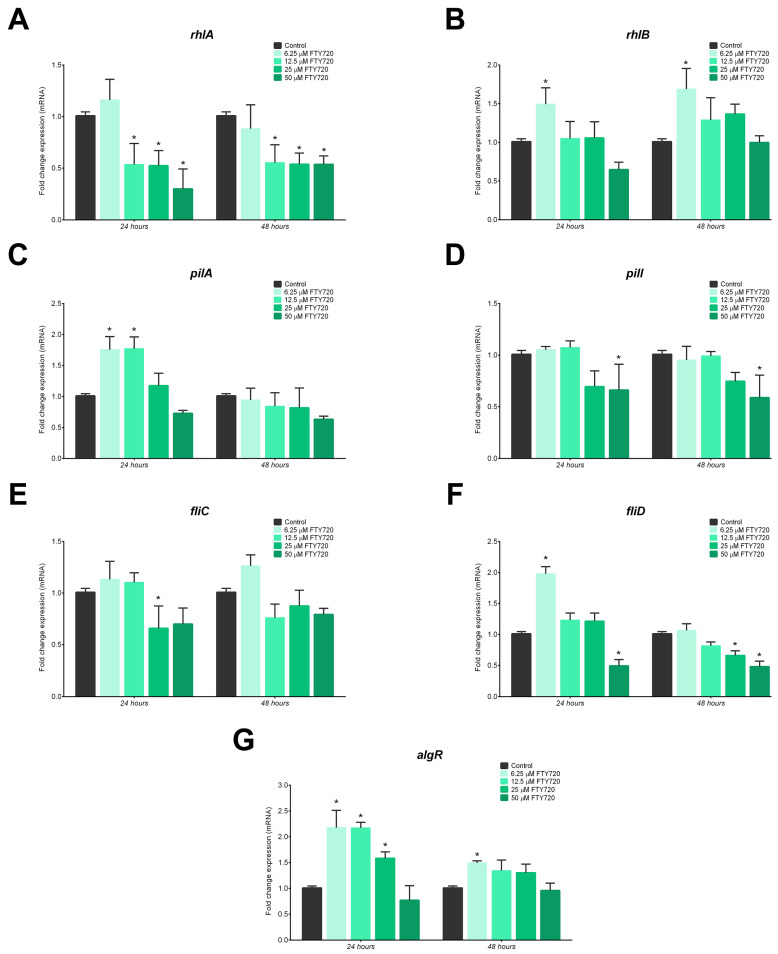
Effects on the expression levels of selected genes involved in the motility and biofilm formation of *P. aeruginosa* after 24 and 48 h exposure to FTY720. Expression of (**A**) *rhlA* and (**B**) *rhlB* for rhamnolipid production; (**C**) *pilA* and (**D**) *pilI* for pilin; (**E**) *fliC* and (**F**) *fliD* for flagellar filament; and (**G**) *algR* for alginate biosynthesis. Data are presented as mean fold change ± SD (n = 9); * *p* < 0.05.

RT-qPCR was performed to detect the expression of genes involved in biofilm formation. *rhlA* and *rhlB* are responsible for rhamnolipid production. *rhlA* showed a significant reduction in gene expression at 12.5 and 25 µM FTY720 when exposed for 24 h and 48 h (Figure 9A). *rhlB*, on the other hand, showed increased expression at 6.5 µM FTY720 at both exposure times when compared to the control (Figure 9B). *pilA*, a major pilin, showed significant overexpression of about 76% after 24 h for both 6.25 µM and 12.5 µM (Figure 9C), whereas *pilI* expression was significantly reduced at a 50 µM dosage for both incubation periods (Figure 9D). For the *fliC* expression, which relates to flagellar filaments, a significant decrease was observed at the 25 µM concentration (Figure 9E). Treatment with 50 µM FTY720 did not significantly affect gene expression. Another gene related to the flagellum, *fliD*, displayed significantly increased gene expression after 24 h of exposure to 6.25 µM FTY720; however, it decreased after 48 h (Figure 9F). FTY720 at a concentration of 12.5 µM slightly decreased *fliD* expression, whereas a concentration of 25 µM induced a significant reduction in gene expression after 48 h of exposure. Treatment with 50 µM FTY720, on the other hand, significantly downregulated *fliD* expression at 24 and 48 h of exposure. *algR*, required for alginate biosynthesis, showed an increased gene expression at 24 h as the FTY720 doses decreased (Figure 9G). After 48 h of treatment, only 6.25 µM FTY720 induced a significant increase in expression as compared to the control group.

## 3. Discussion

The present study investigated the efficacy of fingolimod hydrochloride at concentrations of 3.13 to 400 µM against *Pseudomonas aeruginosa*, focusing on the effects on antibacterial activity, virulence factor production, biofilm inhibition, and viability assays.

In this study, the MIC of FTY720 against *P. aeruginosa* PAO1 was determined to be 100 µM. The viability assay confirmed a decrease in the growth of *P. aeruginosa* PAO1 cells upon exposure to FTY720. Based on these results, it can be seen that the reduction in bacterial cell activity increases over longer exposure times, wherein 48 h of treatment resulted in an additive effect that resulted in further reduction in bacterial activity. Although FTY720 is considered to be a hydrophobic compound, it is possible that the compound can penetrate the outer membrane bilayer of *P. aeruginosa* through the self-promoting pathway, which further exerts its antibacterial effects [38].

Our results further reveal that increasing FTY720 in the culture medium significantly inhibited PAO1 biofilm formation in a dose-dependent manner. This could be due to fingolimod directly affecting biofilm integrity in the extracellular matrix [39]. In contrast, fingolimod ≥ 100 µM has been reported to have a minimal effect against specific *P. aeruginosa* strains, such as controlling planktonic development without directly affecting biofilm formation [40]. Moreover, the different susceptibilities of *P. aeruginosa* strains and the differing experimental conditions in terms of the growth medium and parameters under which biofilms were generated might explain these varied activities [41]. In the case of *A. baumannii* biofilms, 25 μM FTY720 demonstrated effective inhibitory activities in contrast with those obtained for *P. aeruginosa* PAO1 [40], which can likely be attributed to different stress and adaptive responses of Gram-negative bacteria, wherein different functions are regulated or activated [42,43]. Despite this, 12.5 μM FTY720 still showed significant inhibitory effects against biofilm formation and virulence factor production in *P. aeruginosa*, with no effect on the cellular viabilities of human cell lines. The results also showed that 6.25 µM FTY720 disrupted the biofilms of *P. aeruginosa* without directly affecting bacterial growth, suggesting its specificity in targeting the biofilm formation and structure of *P. aeruginosa* without impacting its overall growth. This unique mechanism may decrease the risk of resistance developing in *P. aeruginosa* biofilms and can possibly be used in future combination therapy with antibiotics [44,45]. 

The results also demonstrated that pigments biosynthesized by *P. aeruginosa* PAO1 were affected in a significant manner by FTY720 treatment after testing various concentrations. This agrees with previous studies wherein reduced pyocyanin production mediated the generation of thick biofilm aggregates containing extracellular DNA that are important in the pathogenesis of *P. aeruginosa* [46,47]. Furthermore, the changes in the amount of pyoverdine synthesized with 12.5–50 µM FTY720 showed a strong correlation with inhibited biofilm formation. This could be due to the mechanism of fingolimod inducing growth environments which can lead to selective pressures that influence reduced biofilm densities [48]. Together, these results show that modulating pigment production can potentially impact the early stages of *P. aeruginosa* biofilm formation.

The results of RT-qPCR revealed that rhamnolipid-associated (*rhlA*, *rhlB*), pilus assembly (*pilA*, *pilI*), and flagellum assembly (*fliC*, *fliD*) genes were all significantly downregulated after treatment with 12.5–50 µM FTY720, while the gene related to alginate synthesis (*algR*) was not [49,50,51,52]. Adherence ability and motility are also essential properties for biofilm development, and therefore, the downregulation of motility genes plays an important role in the adherence and antibiofilm potential of *P. aeruginosa*. The mechanism of action for FTY720 on bacteria has not been fully established and has not been reported yet. This work is one of the first papers to report on the potential mechanisms of action against biofilm formation through the downregulation of two important biofilm genes, *rhlA* and *fliD*. Considering the important role of motility in regulating *P. aeruginosa* biofilm development, the results suggest that the FTY720-mediated inhibition of biofilm formation and virulence factor production is correlated with these factors and may be further developed to control *P. aeruginosa* biofilm infections [44,53,54].

At a concentration of 6.25–50 µM, the analysis indicated that FTY720 may be a negative regulator of biofilm formation at a low concentration based on the overall trend. This adaptive response has been shown previously by Kaplan et al. for low concentrations of β-lactam antibiotics, which induced aggregation and biofilm formation in *S. aureus* [55]. Similar findings were observed by Rodríguez-Melcón et al., wherein low doses of sodium hypochlorite induced biofilm formation in *Listeria monocytogenes* [56]. Similarly, Oliveira et al. reported that antibiotics at low concentrations induce the formation of biofilms in *P. aeruginosa* [57], which further supports the up-regulation trend observed in the mRNA levels produced by 6.25 µM FTY720. Indeed, it is commonly observed in various types of bacteria, wherein low doses of antibiotics can induce the defensive mechanism of sub-inhibitory stress, which may promote the increased production of extracellular polymeric substances in the biofilm matrix and induce biofilm formation [56].

It is worth noting that previous work on the effects of fingolimods on microorganisms demonstrated results supporting the use of these drugs as growth inhibitors due to their direct effect on preventing bacterial growth without significant negative effects on human cells [58,59]. Their results showed that the sub-inhibitory concentrations of FTY720 at 25–50 μM exhibited a significant amount of cytotoxicity on normal human cell lines. Najarzadegan et al. reported that FTY720 effectively inhibits *C. albicans* growth and can be possibly repurposed as a clinical antifungal agent [34]. An interesting study by Zore et al. developed a library of 28 fingolimod derivatives and evaluated their antibacterial and antibiofilm activities [22]. They were able to show that recently identified fingolimods are potent antibiofilm compounds. They also showed that seven derivatives were more effective against *S. aureus*, while five other derivatives showed improved bioactivities against *A. baumannii* and *P. aeruginosa*, with no effect on human cell viabilities. The varied antibacterial activities of the different fingolimod analogs can also be attributed to modifications in chemical structure which can directly disrupt the Gram-positive cell wall structure more effectively. Gilbert-Girard et al. determined that fingolimod displayed bactericidal activity against some strains, and reported the inhibitory activity of sphingosine analogs against *C. violaceum* [40]. Another study by Geng et al. showed that fingolimod effectively reduced biofilm formation, exopolysaccharides, motility, and bacterial abundance within *K. pneumoniae* biofilms without impeding growth and metabolic activity [28]. This finding is contradictory to what was found in this work, where redox potential for *Pseudomonas aeruginosa* was significantly reduced in addition to its antibiofilm effects.

The data have demonstrated that fingolimod hydrochloride has an effect on the viabilities of hMSC-TERT2 20 and HGNF cells in a dose-dependent manner, wherein direct cytotoxicities were observed at 25–50 µM. This study is the first evaluation of the cytotoxic effects of fingolimod on human mesenchymal stem cells. To our knowledge, the effect of FTY720 on normal cell lines has not been fully explored, and few studies have examined how FTY720 promotes the proliferation and migration ability of normal neural stem cells [60,61]. Neural progenitor cells have very limited self-renewal and proliferative capacities as compared to mesenchymal stem cells, and therefore exhibit varied viabilities [62]. 

These results warrant additional investigation to further explore the underlying mechanisms and specific effects of fingolimod hydrochloride on *P. aeruginosa* growth and biofilm inhibition. Additional research is needed to fully evaluate its efficacy in different models and clinical settings, which may support its use as an antimicrobial agent in the future.

## 4. Materials and Methods

### 4.1. Bacterial Strain, Culture Conditions, and Chemicals

*Pseudomonas aeruginosa* PAO1 [63] was kindly donated by Prof. Lee Hughes from the University of North Texas, Denton, Texas. *P. aeruginosa* PAO1 cells were cultured on a nutrient agar plate (Oxoid, Hampshire, UK) for 24 h at 37 °C. To prepare the bacterial culture, a single isolated colony was inoculated in 10 mL of nutrient broth (NB; Oxoid) and incubated for 24 h at 37 °C in the Excella E24 incubator shaker (New Brunswick Scientific, Edison, NJ, USA). The bacterial cell count was measured by a spectrophotometer (Libra S22, Biochrom Ltd., Cambridge, UK) and standardized at OD600 = 0.5 A (mid-log phase); whereas NB was used for bacterial cultures in all experiments unless otherwise stated.

FTY720 (Selleck Chemicals, Houston, TX, USA; Cat. No. S5002) solutions were prepared in dimethyl sulfoxide (DMSO) and subsequently diluted in fresh NB to obtain the indicated concentrations, and the control group contained an equivalent concentration of DMSO.

### 4.2. Resazurin-Based Turbidometric Assay and MIC Determination 

The resazurin-based turbidometric assay offers a simple, rapid, and sensitive measurement for the inhibition effects on bacterial activity of FTY720 against *P. aeruginosa* for the minimum inhibitory concentration (MIC) determination. This assay was conducted based on the protocol as previously described [64,65]. Eight two-fold serial dilutions of FTY720 ranging from 3.13 to 400 µM were prepared in wells of a 96-well round-bottom plate. Gentamicin 1 µM was added as a positive control, 0.2% DMSO was added as a vehicle control, and NB was added as broth sterility control. Broth microdilutions were performed precisely according to the Clinical and Laboratory Standards Institute (CLSI) protocol [66]. After this, 50 µL of a diluted PAO1 bacterial suspension in NB at an initial turbidity of 0.50 A (OD600) was added to the test wells accordingly, and then incubated at 37 °C for 24 h. Different control groups were also included, such as NB as the sterility control, gentamicin as the antibiotic control, and PAO1 as the growth control. After incubation, 20 µL of 0.015% resazurin working solution (Sigma-Aldrich, St. Louis, MO, USA) was added to all wells and incubated at 37 °C for 4 h. Finally, fluorescence was measured using a Synergy HT microplate reader (BioTek Instruments, Winooski, VT, USA) at excitation and emission wavelengths of 530 and 590 nm, respectively. The MIC was determined as the lowest drug concentration that led to no visible color change in the broth and inhibited bacterial growth.

### 4.3. Cytotoxicity Studies on Human Cell Lines

The effects of FTY720 on the cell proliferation of normal human cells were assessed using the Alamar Blue assay [67,68]. Human bone marrow-derived mesenchymal stem cells that were immortalized via the genetic overexpression of telomerase reverse transcriptase [62] were kindly provided by Prof. Moustapha Kassem from the University of Southern Denmark, Odense, Denmark. Primary human gingival fibroblasts (Catalog# 2620, ScienCell Research Laboratories, Carlsbad, CA, USA), isolated from human gingiva, were also used in this study. Both cell lines were cultured in Dulbecco’s modified Eagle’s medium (DMEM) supplemented with 10% fetal bovine serum, 1% penicillin–streptomycin, and 1% non-essential amino acids (all from Gibco, Invitrogen, Carlsbad, CA, USA) at 37 °C and 5% CO_2_ in a humidified incubator.

Cells were seeded at a density of 5 × 10^4^ cells in 100 µL of DMEM per well in 96-well culture plates which were grown to a sub-confluent monolayer for 24 h. After medium change, the confluent cultures were then treated with the FTY720 compound at concentrations of 3.13, 6.5, 12.5, 25, and 50 µM in 0.2% DMSO for 24 and 48 h. The negative control consisted of cells treated with the vehicle, DMSO. After the specified incubation time, 10% (*v*/*v*) Alamar Blue reagent (AbD Serotec, Raleigh, NC, USA) was added to the wells and incubated for 4 h at 37 °C. The fluorescence intensities were measured at λ_ex_ = 560 nm and λ_em_ = 590 nm using a BioTek Synergy HT plate reader, and the cell viability percentages were calculated based on cell viability % = 100 × (FI 590 of the cells treated with FTY720 ÷ FI 590 of untreated control cells), where FI 590 = fluorescent intensity at 590 nm emission (560 nm excitation).

### 4.4. Growth Curve

The FTY720 concentrations used for further experiments were selected based on preliminary MIC experiments.

A growth curve for PAO1, cultivated in the presence or absence of FTY720, was used to investigate the potential effects on bacterial growth, as previously studied [69]. Bacterial inoculates were grown overnight and, at an initial turbidity of 0.10 A (OD600), were added to the wells of a 96-well microtiter plate containing two-fold serial dilutions of FTY720 ranging from 3.13 to 100 µM in 100 μL of NB. Control sample wells were loaded with 100 µL of NB only, instead of FTY720. The plates were sealed and incubated at 37 °C while shaking, and the OD600 was recorded using a BioTek Synergy HT microplate reader at 15 min intervals for up to 20 h.

### 4.5. Biofilm Inhibition Assay

The effects of FTY720 on biofilm mass formation were assessed using a microtiter plate-based method in 96-well U-shaped bottom polystyrene plates as previously described [70]. Briefly, PAO1 cells were inoculated in NB at an initial turbidity of 0.50 A (OD600) and were cultured with or without FTY720 doses (6.25–50 µM) for 24 h at 37 °C. A negative control consisting of fresh medium without any bacterial inoculum was included. After incubation, nonadherent bacteria and excess media were removed by washing the 96-well plate repeatedly with sterile distilled water and left to dry for 20 min. Thereafter, 150 µL of 0.5% crystal violet solution (Sigma-Aldrich) was added into each well. The plates were left at room temperature for 10 min and subsequently repeatedly submerged into distilled water to remove excess dye. After drying, 200 µL of 100% ethanol (Sigma-Aldrich) was added into the wells and incubated at room temperature for 15 min. The absorbances of the dye were measured spectrophotometrically at 490 nm with a BioTek Synergy HT microplate reader.

### 4.6. CLSM Analysis of Biofilm Morphology

The effects of FTY720 on biofilms of *P. aeruginosa* were further assessed with confocal laser scanning microscopy (CLSM). This assay was conducted as previously described [71,72]. PAO1 cells were inoculated into 2 mL of NB medium at an initial turbidity of 0.50 A (OD600) and were cultured on coverslips in 6-well culture plates at 37 °C. After 24 h of bacterial colonization, 2 mL of NB was added to each well with or without FTY720 concentrations and incubated for an additional 48 h. Thereafter, the remaining broth was removed from each well and nonadherent cells were removed by washing thrice with phosphate-buffered saline (PBS). The coverslips were carefully transferred to a new 6-well plate and about 200 µL of LIVE/DEAD™ BacLight™ working solution (Invitrogen Ltd., Paisley, UK) was added onto each coverslip using the manufacturer’s instructions and then incubated for 30 min at room temperature in the dark. The morphology of PAO1 biofilms developed without or in the presence of FTY720 was imaged with Nikon C2 CLSM (Nikon Instruments Inc., Tokyo, Japan). The excitation/emission was 488 nm/<550 nm for SYTO^®^ 9 (BacLight™ Component A) and 568 nm/>600 nm for propidium iodide (BacLight™ Component B). The images were captured, and the three-dimensional plots of biofilm samples were constructed with NIS-Elements Advanced Research Software (version 4.0, Nikon, Japan) [73]. The image analysis method was carried out using ImageJ software (version 1.50i, NIH) [73]. The death percentage of PAO1 cells was calculated by dividing the red fluorescence (dead bacteria) by the green + red fluorescence in each acquired image (live and dead bacteria), then multiplying this value by 100, as previously described [74]. The inhibitory effect of different concentrations after treatment on biofilm depth and formation were visualized and evaluated on the generated sample optical slices [75].

### 4.7. Pigment Production

#### 4.7.1. Pyocyanin Assay

The assessment of the inhibitory effect of FTY720 on the production of the bluish-green pyocyanin pigment by *P. aeruginosa* was performed using King’s A broth, the reference medium to stimulate pyocyanin production [76,77]. PAO1 overnight cultures were adjusted to 0.50 A (OD600), and 10 μL aliquots of the bacterial suspensions were grown in 1 mL of King’s A broth [77] with or without FTY720 treatment overnight at 37 °C with shaking. Absorbance readings of the produced pyocyanin were measured at 600 nm using a Biochrom Libra S22 spectrophotometer. Then, 1 mL of the pyocyanin dye mixture was transferred to a 15 mL conical tube and centrifuged for 25 min at 1800× *g* using a HERMLE Z 206 A centrifuge (Wehingen, Germany). The supernatant was then filtered into a 15 mL conical tube using a 0.45 µm syringe filter and 600 µL of chloroform (Sigma-Aldrich) was added to precipitate organic matter followed by vortexing. The resulting mixture was centrifuged for 7 min at 1800× *g*; the green top layer was removed and 300 µL of 0.2 N HCl was then added and vortexed. The mixture was recentrifuged according to the same parameters previously mentioned and the upper pink layer was transferred to a new 96-well plate and measured at 520 nm using Synergy HT microplate reader.

#### 4.7.2. Pyoverdine Assay

The quantification of pyoverdine concentration in PAO1 cultures treated with FTY720 was measured using a quantitative chemical assay [76]. Four concentrations of the two-fold serial dilution of the FTY720 drug (6.25–50 µM) were added to King’s B broth [77], which was used to induce pyoverdine biosynthesis. After the standardized PAO1 culture was added, the treated cultures were incubated at 37 °C for 12 h with continuous shaking. Upon incubation, absorbance readings of the cell density were measured at 600 nm. The broth samples were centrifuged at 13,552× *g* for 2 min. Thereafter, 1 mL of cell-free supernatant from the drug-treated or untreated PAO1 cultures were centrifuged at 10,000× *g* using an Eppendorf 5418 centrifuge (Hamburg, Germany). The supernatant was then collected and transferred to a 96-well culture plate and absorbances were quantified at 405 nm using a Synergy HT microplate reader. 

### 4.8. RT-qPCR Analysis

Real-time quantitative PCR was used to investigate the expression of biofilm-related genes of *P. aeruginosa*. The target genes used in this study (Table 1) were determined to be associated with the motility of *P. aeruginosa*, as described previously [78]. PAO1 cells were grown in LB medium at an initial turbidity of 0.50 A (OD600) with shaking at 220 rpm for 24 h, and then cultured in a 6-well culture plate with four concentrations of two-fold serial dilutions of FTY720 (6.25–50 µM) at 37 °C for either 24 or 48 h with agitation. Cells were collected after the specified incubation time and 1 mL of inoculated broth was transferred to an Eppendorf tube and centrifuged at 10,053× *g* for 5 min. After pellet collection, total RNA extraction was performed using HiGene™ Total RNA Prep kit (BioFACT Co. Ltd., Daejeon, Korea) in accordance with the manufacturer’s protocols. The quality and concentration of the extracted total RNA were measured using BioSpectrometer^®^ basic (Eppendorf, Germany). 

Total RNA was reverse-transcribed using a HyperScript™ RT Master Mix (GeneAll Biotechnology Co., Ltd., Seoul, Republic of Korea) and a GeneAmp™ PCR System 9700 thermal cycler (Applied Biosystems, Carlsbad, CA, USA) according to the manufacturers’ instructions.

Fluorescence real-time quantitative PCR was performed with 5× HOT FIREpol^®^ EvaGreen^®^ qPCR Supermix (Solis BioDyne, Tartu, Estonia) using an ABI 7500 Real-Time PCR System (Applied Biosystems, Waltham, MA, USA). The following reaction conditions were used: 94 °C for 12 min, followed by 40 cycles of 95 °C for 15 s, 65 °C for 30 s and 72 °C for 30 s. The *16S rRNA* was chosen as an internal control for normalization and to calculate the relative fold changes in gene expression using the 2^−ΔΔCT^ method [79]. The experiments were repeated independently three times using different RNA samples.

### 4.9. Statistical Analysis

The results are expressed as the mean ± standard deviation (SD) and represent the data obtained from at least three independent experiments in triplicate. All statistical analyses and graphs were performed using GraphPad Prism (version 6.00 for Windows, La Jolla, CA, USA; https://www.graphpad.com). Depending on the experiments, one-way analysis of variance followed by Dunnett’s post hoc test or two-way analysis of variance followed by Bonferroni’s post hoc test were used for analysis. Differences with *p* ≤ 0.05 were considered statistically significant. 

## 5. Conclusions

In conclusion, this study demonstrated the potential of FTY720, an S1PR modulator for multiple sclerosis treatment, to significantly reduce the biofilm, virulence, and bacterial viability of *Pseudomonas aeruginosa*. This drug could be an alternative agent to combat biofilm-related infections. However, further studies on the mechanisms of antibiofilm properties as well as in vivo models and clinical settings are necessary to confirm these findings.

## Figures and Tables

**Table 1:** Genes selected and their primer sequences.

Genes	Primer Sequence (5′-3′)
*rhlA*	F: GGC GAT CGG CCA TCT G R: AGC GAA GCC ATG TGC TGA T
*rhlB*	F: GCC TGT CGG CGT TTC ATG R: CAT CCC CCT CCC TAT GAC AA
*pilA*	F: TGC GCG TTC GGA AGG T R: CGA CTC TTC AAC AGT GGT CTT CA
*pilI*	F: GCA CTG CAA CCC TTC ATT CAT R: CGC ATG CGG GCT GAA C
*fliC*	F: CAG TGC CAA GGA CGA TGC T R: AAC GTT CAG ACC GCT GAT CTG
*fliD*	F: TGG CTG GCA CCT ACC AGA TC R: GGC CTG GAG GGC AAT CTT
*algR*	F: CCT CGG CCA GCA ATG G R: CGG ATA TCC AGC AGG ACG AT
*16S rRNA*	F: GCGCAACCCTTGTCCTTAGTT R: TGT CACCGGCAGTCTCCTTAG

## Data Availability

All data generated during this study are presented in this paper.
